# Single‐Molecule Imaging and Spectroscopy Enables Quantification of Location‐Dependent Light–Matter Interactions on Nanoantennas

**DOI:** 10.1002/smsc.202500597

**Published:** 2026-03-05

**Authors:** Lukas Lang, Sjoerd Nooteboom, Teun A. P. M. Huijben, Sarojini Mahajan, Rodolphe Marie, Peter Zijlstra, Monika Fleischer

**Affiliations:** ^1^ Institute for Applied Physics and Center LISA^+^ Eberhard Karls University Tübingen Tübingen Germany; ^2^ Department of Applied Physics and Science Education Eindhoven University of Technology Eindhoven The Netherlands; ^3^ Department of Health Technology Technical University of Denmark Lyngby Denmark

**Keywords:** localization microscopy, nanoantenna, nanophotonics, plasmonics, point‐spread function, single‐molecule imaging, single‐molecule spectroscopy

## Abstract

Individual dye molecules coupled to a plasmonic nanoantenna have been established as a versatile foundation for single‐molecule studies. Crucial parameters such as fluorescence intensity enhancement, spectral properties, and emission patterns sensitively depend on the exact position of the dye molecule relative to the antenna and its hot spots. Knowledge about the binding location is therefore of paramount interest, however, it is highly challenging to obtain. We present a comprehensive approach based on correlative microspectroscopy of the optical properties of single fluorophores that transiently bind to gold nanocones using the DNA‐PAINT method. These 3D nanoantennas offer independently tunable in‐ and out‐of‐plane plasmon resonances with strong electric field enhancements at the tip apex and the nanocone base. We exploit site‐specific deformations of the point spread function in a high‐throughput approach as a means to correlate the position of the fluorophores on the nanoantenna surface to previously inaccessible parameters, investigating location‐specific binding probability, mode‐dependent spectral reshaping, and location‐resolved fluorescence enhancement factors. Our approach provides unprecedented multimodal quantification by correlating spatial and spectral information to open new avenues in fundamental studies of light–matter interactions and applications like biosensing.

## Graphical abstract

The fluorescence intensity as well as the spectral shape of the emission of single dye molecules strongly depend on the molecules’ exact position relative to plasmonic nanoparticles in hybrid systems. By point‐spread function modification analysis, the vertical binding location of single fluorophores on gold nanocones is obtained and correlated to both spectral features and particle‐size dependent binding rates.

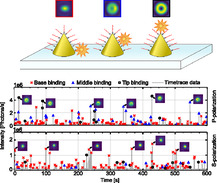

## 1 Introduction

Plasmonic noble metal nanoparticles are well established for providing unique optical properties that are being utilized in manifold applications, for example, for optical antennas, single‐molecule sensing, and Raman enhancement [1, 2, 3]. Localized surface plasmon resonance (LSPR) modes that refer to collective oscillations of the conduction electrons in the particles can be directly excited by incident electromagnetic radiation. Due to the accumulation of charges at the particle surface, further enhanced by the lightning rod effect, strong and highly localized evanescent electric fields are generated. These so‐called hot spots are confined to volumes much smaller compared to conventional diffraction‐limited focal spots. The spectral position of these enhanced electric near fields can be tuned by the geometry of the particles themselves, thus directly influencing the LSPRs [4, 5]. Antennas with distinguishable in‐plane and out‐of‐plane LSPRs can be realized by crafting three‐dimensional nanostructures. This is demonstrated with gold nanocones that offer two orthogonal dipolar LSPR modes that can directly be excited by suitable polarization of the incident light [6]. Electric field components parallel to the vertical nanocone axis excite an out‐of‐plane mode that provides a localized and strongly enhanced electric hotspot at the tip apex. In contrast, polarization parallel to the substrate surface excites an in‐plane mode along the base of the nanocone. Through the nanocone geometry, both modes can be spectrally detuned to an adjustable extent [6, 7, 8].

The properties of plasmonic particles are widely exploited in, for example, surface‐enhanced Raman spectroscopy techniques, label‐free sensing applications that are based on the dependence of the LSPR position on the surrounding dielectric environment, as well as assays based on the detection of fluorescence [3, 9, 10, 11]. In the latter sense, coupling of fluorescent emitters to plasmonic nanoantennas is of high interest for studying light–matter interactions on a single‐molecule level [1, 2, 12, 13, 14]. Single‐molecule investigations are paramount for accessing molecular properties that are lost through averaging in ensemble studies. In pioneering work, it was shown that single molecules may be detected and traced [15, 16, 17], however their intensity is very low, such that enhancement mechanisms are desirable [18, 19, 20]. A general strategy to utilize fluorescence detection in single‐molecule sensing is to induce transient fluorophore binding through molecular recognition. Here, DNA‐mediated points accumulation for imaging in nanoscale topography (DNA‐PAINT) can be applied [21]. This method is widely used in biosensing and additionally offers the possibility for sequence‐specific DNA detection [22, 23]. In the vicinity of metal nanoparticles, the emission properties of single emitters are strongly altered as a consequence of modifications in the fluorophore's decay rates as well as increased excitation rates due to the local field enhancement [4, 13, 24]. Thus, the emitted fluorescence intensity may be either increased or quenched depending on the distance between the molecule and the particle's surface [1, 25]. It was also shown that the spectral shape of the fluorescence emission can be modified due to the dispersive nature of the radiative and nonradiative decay rate modification, as has been demonstrated for single nanoparticles and dimers [26, 27, 28, 29]. Ultimately, the positioning of the emitter in the electric field hot spots determines the fluorescence signal strength and the feasibility of single‐molecule detection. Moreover, the emitter position relative to the particle dictates the modification of the fluorescence properties. Thus, a determination of the binding position is crucial, however remains extremely challenging. In fluorescence assays, nanospheres or nanorods are predominantly used due to their facile preparation. For these systems, effort has been put into 2D binding‐position localization for super‐resolution imaging [30, 31, 32], which was recently extended to a 3D localization for spherical systems by the authors [33]. Still, spherical particles are hampered by mode degeneracy and lower mode confinement, while the positioning of hot spots close to the interface may lead to unwanted substrate effects like, for example, hindered analyte diffusion or remaining background fluorophore excitation in methods relying on total internal reflection illumination. Therefore, it can be beneficial to move the hot spots farther away from the substrate using dedicated nanostructure geometries. By crafting nanoantennas with distinguishable in‐plane versus out‐of‐plane LSPRs, additional information on the binding positions can be gained.

In this work, we address these challenges by exploiting the optical properties of conical gold nanoantennas in single‐fluorophore binding studies utilizing a DNA‐PAINT approach. This allows us to reveal the binding location of dye molecules at the single‐molecule level with nanometric spatial precision. By correlating the point‐spread function (PSF) distortions, measured intensities, and spectral properties of individual binding events, position‐dependent characteristics of the fluorophore emission and binding probabilities are extracted and compared to theory by extensive modeling. This correlative approach thus offers a strategy for linking high‐resolution 3D binding‐position localization to observed properties, where conventional localization techniques based on 2D Gaussian fitting might be prone to mislocalizations [31]. Consequently, for example, site‐specific binding kinetics may be investigated, offering a path toward otherwise inaccessible parameters such as locally modified diffusion conditions.

## 2 Results

### 2.1 Measurement Setup

**FIGURE 1:**
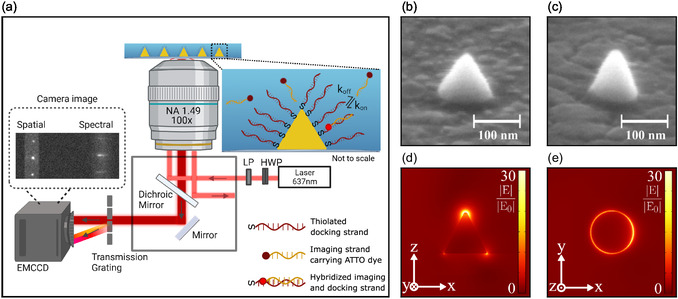
(a) Schematic overview of the total internal reflection setup. A 637 nm laser, a lambda‐half waveplate (HWP), and a linear polarizer (LP) provide polarized excitation. Spatial and spectral information on the emitted fluorescence signal is imaged on the camera upon DNA hybridization at the gold nanoparticles. (b,c) SEM images of fabricated gold nanocones on glass/ITO substrate prepared with (b) AR ≈ 1 and (c) AR > 1 for a viewing angle of 60° (an overview for all fabricated nanocone sizes is found in Figure S1). (d) Vertical and (e) horizontal cross sections of FEM simulations of the electric near‐field enhancement around the nanocone on ITO/glass immersed in water using p‐polarized excitation (in (e) the cross section is taken on the substrate interface). Strong field enhancement is achieved at the nanocone tip and the base.

In the DNA‐PAINT method applied here, the hybridization of two complementary, single‐stranded DNA molecules is monitored through the use of an ATTO 655‐labeled ssDNA imager. Here, the imager strand carries the dye molecule, while the docking strand is bound to the nanocone. Experimentally, a total internal reflection fluorescence setup is employed, as is schematically visualized in Figure 1a (see also Section 4). The setup allows for polarized evanescent excitation of the nanocones through their one‐photon photoluminescence as well as the freely diffusing imagers with a high signal‐to‐background ratio. The total fluorescence containing information on the PSF is imaged on an EMCCD camera, while single‐molecule emission spectra are obtained by placing a diffraction grating before the camera. To investigate the effect of the optical properties of the nanoantenna, we compare nanocones with varying geometry and thus different plasmonic properties. Gold nanocones of different sizes and aspect ratios (ARs) with high structural uniformity are fabricated on a glass substrate by transferring the pattern of an aluminum oxide etch‐mask into a continuous gold film (see Section 4 for details). Exemplary scanning electron microscopy (SEM) images of fabricated structures with different ARs are shown in Figure 1b,c. Arrays of gold nanocones with AR ≈ 1 and coinciding tip and base resonances (in the following denoted as ‘CR nanocones’ for ‘coinciding resonances’) are fabricated in heights (*H*) and diameters (*D*) ranging from *H* = 88.8 ± 5.6 nm to *H* = 125.1 ± 6.5 nm (see Figure S1). Additionally, nanocones with AR ≈ 1.1, *H* = 110.3 ± 12.8 nm, and *D* = 99.4 ± 5.7 nm are manufactured. Their differences in geometry give rise to spectrally separable resonances (the particles are hence denoted as ‘SR nanocones’ for ‘separable resonances’). ‘Spectrally separable’ is defined here as the tip and base resonance maxima being resolvable by curve fitting since they are separated by >50 nm in corresponding scattering spectra. For CR nanocones, both resonances are not spectrally distinguishable by dark‐field spectroscopy. Particle sizes are chosen such that their corresponding LSPR wavelengths are in the visible light regime and provide resonances with varying overlap with the used fluorophore emission spectra (see Table S1 for an overview). Numerical simulations are performed to estimate suitable particle sizes. As discussed earlier, the nanoparticles focus the electromagnetic energy at boundaries with large curvatures, that is, predominantly the tip and base edges of the nanocone. By using p‐polarized evanescent excitation, a combination of the plasmonic tip and base resonance is excited, leading to 1–2 orders of magnitude enhancements of the electric near field at either position. In contrast, only the in‐plane base resonance is excited using s‐polarization. Figure 1d,e shows finite element method (FEM) simulations of the electric near‐field distribution around gold nanocones immersed in water for p‐polarized total internal reflection excitation (see Figure S2 for a comparison to s‐polarized excitation).

The functionalization and single‐molecule imaging of the gold nanocones is performed following previous works as described in further detail in Section 4 [13, 34]. Briefly, the nanocones are first functionalized by a thiolated docking strand whose oligomeric DNA sequence is complementary to that of the imaging strand. The imaging strands are fluorescently labeled with either ATTO 655 or ATTO 643. They bind transiently to the docking strands with a typical bound‐state lifetime of ∼1 s, as their melting temperature is chosen as 22°C, and the measurements are conducted in solution at room temperature. The concentration of the imager strands is sufficiently low (1 nM) to achieve at most one imager bound to a particle at any time. Once bound, the distance of the fluorophores to the nanocones is 3–7 nm as specified by the time‐averaged end‐to‐end distance of the docking strand. Such binding events occur on the whole surface of the nanocone because Brownian motion followed by binding is a stochastic process. The reversibility of the single–molecule interactions enables the collection of a large amount of statistics from sequential binding events. Observation of non‐specific binding events can largely be ruled out, as was demonstrated in ref. [33] when using non‐complementary imaging strands.

### 2.2 Far‐Field Optical Properties of Single Gold Nanocones

**FIGURE 2:**
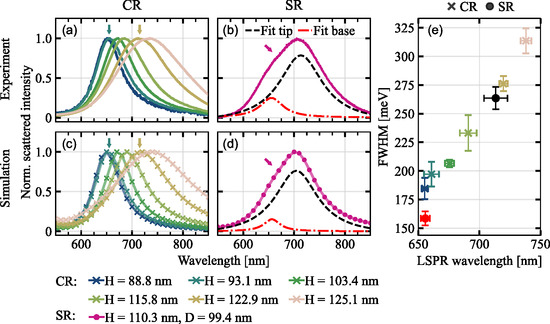
(a) Dark‐field scattering spectra averaged over single‐particle spectra of CR (coinciding resonances, *H* ≈ *D*) gold nanocones with increasing particle size immersed in water under unpolarized excitation (averaged over the individual spectra of six different particles with nominally identical sizes, see Table S1 for particle dimensions). Colored arrows denote particle sizes used in fluorescence reshaping measurements. (b) Averaged dark‐field scattering spectrum of SR (separable resonances) gold nanocones under unpolarized excitation including double‐Lorentzian fits of the tip and base resonance. Arrow indicates that nanocones are also investigated for fluorescence reshaping. (c,d) FEM simulations of the scattered power under dark‐field illumination and detection conditions for comparable nanocone geometries. Arrows denote particle sizes used in fluorescence reshaping simulations. (e) Mean spectral LSPR properties of differently sized CR and SR gold nanocones determined by single‐ or double–Lorentzian curve fitting of the obtained non‐averaged experimental spectra. Error bars correspond to the standard deviation of the fitting parameters (spectra of six individual nanocones are averaged and fitted for each nanocone geometry and used for error estimation).

Single‐particle dark‐field spectroscopy is performed, as the spectral properties determine particle‐fluorophore coupling and resulting modifications of the intensity, PSF, and spectrum of single‐molecule emission. For that, the nanoparticles are immersed in purified water to match the refractive index for the subsequent fluorescence studies. By the use of unpolarized illumination at high angles through a dark‐field condenser, both the plasmonic tip mode as well as the base mode are excited and contribute to the obtained scattering spectra. Examples of spectral features are shown in Figure 2. Gold nanocones with an AR of ∼1 display one dominant LSPR scattering peak as demonstrated in Figure 2a, since the tip and base mode are spectrally overlapping (CR nanocones). By increasing the size of the nanoparticles, the LSPR wavelength is red‐shifted due to the modified particle polarizability in accordance with theory and simulation [5, 35]. For nanocones with an AR > 1, base and tip LSPR wavelengths no longer coincide but instead are spectrally separable by the emergence of a shoulder at the low‐wavelength flank of the dominant resonance (SR nanocones, see Figure 2b). Here, the tip resonance appears at longer wavelengths relative to the base resonance as a result of the different polarizability in the direction of the out‐of‐plane nanocone axis. Larger polarizability leads to stronger scattering that is shifted to longer wavelengths. The spectra are fitted with either single or double Lorentzian functions depending on the number of separable peaks. Figure 2c,d shows the simulated scattering spectra matching the experimental data for further use in complementary simulations (see Section 4 for details on the simulation geometry and parameters). Average fitting results for the LSPR wavelength and full width at half maximum (FWHM) are summarized in Figure 2e. A wide range of plasmonic resonances in the visible to near‐infrared regime are achieved for nanocones with AR ≈ 1, ranging from 655.2 ± 2.6 to 738.0 ± 4.4 nm. Hence, the spectral overlap between the LSPR and the emission wavelength $\lambda_{\mathrm{em}}$ = 680 nm of ATTO 655 can be systematically varied. Notably, the linewidth of the plasmonic resonances increases roughly linearly with the LSPR wavelength, which is due to increased radiation damping for larger particles [35]. The resonances of one investigated geometry are separable and yield on average 655.7 ± 3.6 nm for the base mode and 713.3 ± 9.6 nm for the tip mode. The base resonance overlaps strongly with the emission wavelength $\lambda_{\mathrm{em}}$ = 665 nm of ATTO 643 while the tip resonance falls further into the vibronic tail of the emission spectrum. Tuning the LSPRs with respect to the dye's emission spectrum leads to distinct spectral reshaping. The ATTO 643 dye was chosen in this case to enhance the spectral modifications upon tip‐ or base‐binding.

### 2.3 Binding Position Classification by PSF Imaging

**FIGURE 3:**
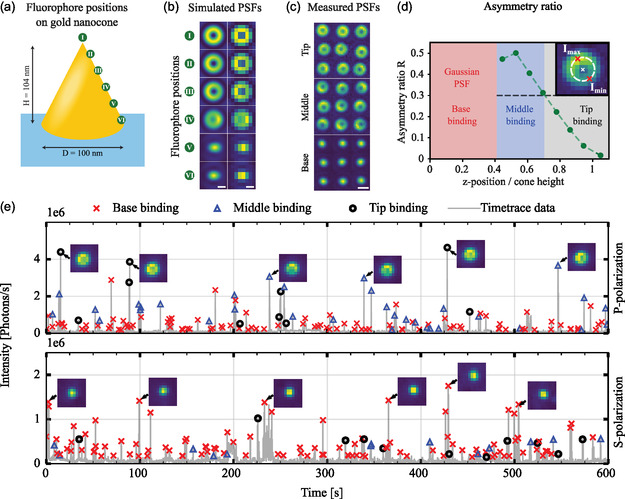
(a) Schematic representation of the considered system with a gold nanocone (*D* = 100 nm, *H* = 104 nm) on top of a glass coverslip coated with a 50 nm layer of ITO. For the PSF calculations, we consider multiple fluorophore positions (green dots) placed 5 nm from the gold surface, equally spaced between the tip and the base of the nanocone (see Figure S3 for details of simulated geometry and fluorophore positions). (b) Numerically calculated theoretical PSFs for the six positions in (a), for a rotationally‐free fluorophore emitting at 675 nm. The left column represents the high‐detail simulated PSFs with a pixel size of 14 nm and the right column the simulated PSFs with a pixel size of 87 nm, the same as in the experiments. PSFs are individually normalized to the brightest pixel (scale bars are 200 nm). (c) Collection of exemplary experimental PSFs obtained in the DNA‐PAINT experiment. The PSFs are classified into three groups based on their shape, representing binding at the tip, middle section, and base of the nanocone (scale bars are 400 nm). (d) Refined calculation of 12 fluorophore positions to determine the binding‐height dependent asymmetry ratio *R*. The inset demonstrates how *R* is calculated. A height ambiguity within the middle section is observed, however still allowing a threefold classification. (e) Exemplary experimental intensity timetraces acquired on a CR nanocone with *H* = 104 nm and LSPR = 676 nm for p‐ and s‐polarized total internal reflection excitation over a time period of 10 min (the intensity is calculated by summing the brightness of 11 × 11 pixels comprising a PSF and converting it to photons). The background luminescence of the gold nanoparticle is subtracted from the data. Tip, middle and base binding events are marked according to the PSF shape imaged on the camera, and exemplary PSF images of the respective events are shown.

In order to resolve the vertical binding position of the fluorescently labeled ssDNA molecules on the nanocone, the PSF of single fluorophores is evaluated. The intensity and shape of the PSF of dipole emitters is affected by their orientation in space, as well as variations of the refractive index in their vicinity given, for example, by plasmonic nanoparticles [36, 37]. The latter is due to interference effects between the dipole emission and secondary sources of radiation induced by polarization changes or currents in the nanoparticle. The typical 2D Gaussian pattern for freely rotating fluorophores in a homogeneous medium [38] is therefore distorted by such coupling with different plasmonic resonance modes. These deformations of the PSF hamper conventional 2D localization microscopy, in which one fits a 2D Gaussian to obtain the fluorophore coordinates, resulting in significant biases or mislocalizations as is widely discussed in literature [30, 31, 39, 40]. The nature of the deformation depends on the geometry of the nanoparticle, the relative positioning between nanoparticle and emitter, as well as the orientation of the latter [36, 41]. Conversely, this deformation can be exploited if the orientation of the dipole emitter is known or freely rotating: comparison of the measured PSF to numerical and/or analytical methods can be used to decode the emitter position relative to the nanoparticle. This was recently shown in the case of spherical nanoparticles by some of the authors [33]. Free rotation of the dipole emitter during binding is hereby assumed, due to the small persistence length of the largely single‐stranded docking strand after binding for the used salt concentration [42]. Corresponding control measurements were performed in ref. [33]. To investigate how the PSF depends on the fluorophore position on the nanocone, we perform numerical PSF calculations [33] (see Section 4 and Figure S3) for multiple emitter positions (Figure 3a). The numerical simulation results show that the PSF for a fluorophore at the tip of the nanocone has a radially symmetric donut shape, while positions in the middle section of the nanocone show asymmetric donut shapes, and the fluorophores at the base of the nanocone an elliptical Gaussian PSF (Figure 3b). Not only does this finding highlight that the shape of the PSF is informative for discriminating between tip‐, middle‐ and base‐binding, it also visualizes which of the two main LSPRs in the nanocone dominates in the coupled system. Donut‐shaped PSFs thus are expected for a coupling to the out‐of‐plane resonance when binding to the nanocone tip, which resembles the analytical image of an out‐of‐plane point dipole. Similarly, coupling to the base resonance dominates upon binding to the nanocone base yielding a Gaussian shape, while a superposition of both is found for binding to the middle section.

In Figure 3c, a collection of experimentally obtained PSFs from the DNA‐PAINT experiment is shown, which closely resemble the numerically calculated ones. In the experiment, the fluorescence intensity bursts due to imager binding and unbinding are monitored on 40–60 nanocones in parallel over a period of 10 min. This is repeated for CR nanocones with increasing heights and diameters. In a first approach, the height‐dependent asymmetry of donut‐shaped PSFs is quantified by the ratio $R=\frac{I_{\max}-I_{\min}}{I_{\min}}$, where *I*_max_ and *I*_min_ are the maximum and minimum intensities of the azimuthal intensity profile respectively at the radius of highest average intensity (see Figure 3d inset). This allows for an estimation of the binding height for events with donut‐shaped PSFs. The correspondence between the ratio *R* and the binding height on the nanocone surface can be extracted from Figure 3d. In the lowest ∼40% of the nanocone height, Gaussian‐shaped PSFs are detected, which are attributed to base binding. Over the top ∼60% of the nanocone height, the extracted ratio *R* can be used to discriminate between tip‐ and middle binding (for the categorization, a threshold of *R* = 0.3 corresponding to the upper third of the nanocone height was chosen). Taking into account the radial direction of the asymmetry, additional information on the azimuthal binding position may be gained, which however is not further pursued here. Exemplary intensity time traces are shown in Figure 3e for p‐ and s‐polarization, and selected PSFs of individual fluorescence bursts are visualized. We observe a mix of both symmetric and deformed donut‐shaped, as well as Gaussian‐shaped PSFs. Tip, middle, and base binding are all detected abundantly for p‐polarization, where the highest intensities of tip binding events generally exceed those for base binding. In contrast, tip and middle binding events are detected only sparsely with strongly reduced intensity for using s‐polarized illumination with a predominantly in‐plane excitation. This is understood as a consequence of the stronger concentration of the electric field enhancement to the nanocone base with only weak electric fields at the tip (cf. Figure S2).

**FIGURE 4:**
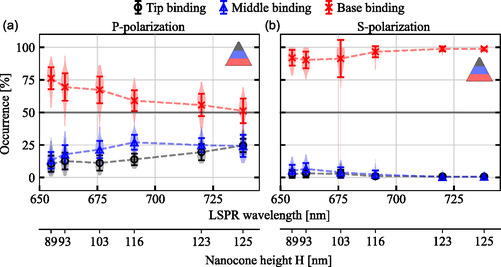
Occurrences of detected tip‐, middle‐ and base‐binding events in percent of the total number of detected events on CR nanocones with increasing size, for (a) p‐polarized and (b) s‐polarized excitation. For the distributions and error calculation, on average 155 binding events on each of ∼50 nanocones per size are evaluated. PSFs with an asymmetry ratio < 0.3 are attributed to binding to the tip, while PSFs with ratio ≥ 0.3 are attributed to binding to the middle section of the nanocone.

Next, we quantify the occurrence of detected binding events for the two different excitation polarizations by classifying the PSFs based on their shape (see Section 4). For p‐polarization, a particle‐size dependent trend of the detected binding occurrence emerges (Figure 4a). From a purely geometrical point of view, the respective accessible surface areas of a nanocone below and above ∼40% of its height, at which the transition from Gaussian to donut‐shaped PSFs occurs (cf. Figure 3d), would equate to ∼64% for base and ∼36% for tip and middle binding, independently of the nanocone size. In contrast, for the smallest nanocones, only around 25% of binding events are detected at the tip and middle section together, whereas 75% stem from the base. The ratio of tip‐ and middle‐ to base‐binding detection then increases with the nanocone size, until ∼50% of occurrences stem from the base for the largest investigated particles and 25% each from the middle and tip. Thus, a simple correlation of the available surface area to the binding probability does not explain the observed trend here. Several other effects may influence the total binding affinity to the tip‐ and middle section compared to the base or the probability of detection at either location. First, hindered diffusion near the water‐substrate interface influences the diffusion coefficients of the imaging ssDNA molecules [43]. Further, numerical studies on the binding probability of molecular agents to plasmonic structures suggest a tendency to bind at sites with high curvatures [44], leading to higher binding rates per accessible surface area. This could be especially relevant for cone‐shaped particles, where the larger accessible interaction volume available for hybridization at the tip enables higher binding rates. An increase of the relative occurrences of tip binding with the particle size further indicates this enlarged probability. In turn, these effects are complemented by site‐specific fluorescence enhancement or quenching that ultimately determine the detectability of binding events, as well as inhomogeneous surface functionalization.

For s‐polarized excitation, strong differences in the detected events are observed compared to p‐excitation (cf. Figure 4b), as the fluorescence enhancement is proportional to the electric field intensity enhancement around the nanoparticle. Here, the majority of the PSFs are attributed to base binding (≳90%), as the electric field hot spots are located mostly at the base of the nanocone, while the fluorescence at the tip is only marginally modified. This also explains the lower correlation of binding localizations with the particle size for s‐polarization. In contrast, for p‐polarization electric fields are enhanced in both areas, and more strongly so at the tip.

For more statistics on the size‐dependent occurrence of Gaussian vs. donut PSF shapes, respectively the trend of observed events under p‐ and s‐polarization, see also Figures S4 and S5.

### 2.4 Location‐Dependence of the Fluorescence Enhancement Factor

The classification into multiple binding areas on the nanocones is exploited to extract position‐dependent modifications of the fluorescence properties of ATTO 655 dyes. We particularly focus on the location‐dependent fluorescence‐enhancement factors (EFs) and the location‐dependent reshaping of the single‐molecule emission spectra.

**FIGURE 5:**
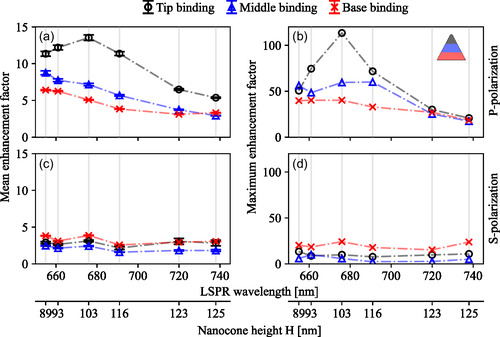
(a) Mean fluorescence intensity EF for tip, middle and base binding events to CR nanocones with increasing sizes under p‐polarized excitation. The mean and error is calculated by averaging over the peak values of the intensity bursts of all binding events at ∼50 particles for each nanocone geometry and binding position and normalizing them to the intensity of non‐enhanced events (for details see main text). (b) Maximum EF as taken from the strongest intensity bursts for tip, middle and base binding under p‐polarized excitation. The values correspond to single events with highest measured intensity. (c,d) same as (a,b) for s‐polarized illumination.

The emitted intensity upon fluorophore binding to the CR nanocones of increasing size is individually tracked over a period of 10 min for 40–60 particles in parallel. From the intensity bursts (i.e. peak values from traces as in Figure 3e), EFs of the emitted fluorescence intensity are calculated for p‐ and s‐polarized excitation to compare the effective enhancement by tip, middle, and base binding. This is done by subtracting the average baseline luminescence in the order of 10^5^ photons/s taken from the time traces with no intensity bursts visible. Normalization is then carried out with the non‐enhanced fluorescence signal of isolated dye molecules that transiently bind (non‐specifically) to the glass‐water interface away from the nanocones. In Figure 5a, EFs for tip, middle, and base binding events are shown as a function of the LSPR wavelength for increasing particle sizes. Here, mean EFs are calculated by averaging over the intensity bursts of all binding events at ∼50 particles for each nanocone geometry. While the mean EFs using p‐polarization (Figure 5a) of base and middle binding display a decreasing trend with the nanocone size and are of similar magnitude (9× to 3×), dyes that bind to the tip are most strongly enhanced on average (14×) at nanocones with $\lambda_{\text{LSPR}}\approx$ 676 nm (∼103 nm nanocone height). In order to estimate the highest possible enhancement, the highest occurring intensity bursts are taken for each particle size and are plotted in Figure 5b. While the described trend is repeated, a maximum enhancement of two orders of magnitude (113×) is obtained for tip binding (additional simulation results are found in the Figure S6a).

The observed fluorescence enhancements result from a combination of several effects. The overall fluorescence enhancement is a combination of an enhanced excitation rate (by the near field around the particle) and an enhanced radiative versus nonradiative decay rate (by coupling of the fluorophore to the dipolar and higher order plasmon modes in the particle). The latter effect results in a modified quantum yield, and thereby either enhancement or quenching depending on the respective magnitudes of the two rates. Following previous work [13, 24, 45, 46], a distinction is made between a weak excitation regime, where the rate of photon emission is directly proportional to the quantum yield and excitation intensity, and a saturation regime, where the photon emission rate scales with the radiative rate of the fluorophore. Thus, for weak excitation, the fluorescence enhancement is maximized when the excitation laser wavelength is resonant with the plasmon. In this regime, higher EFs are expected for the smallest nanocones whose LSPR is closest to the excitation wavelength of 637 nm. This is what we observe for imagers binding to the middle and base of the nanocones in Figure 5a.

In contrast, the highest fluorescence enhancement is achieved for tip binding at an LSPR wavelength of 676 nm, which coincides with the emission maximum of the used ATTO 655 fluorophores. This indicates that the near‐field mediated excitation rate exceeds the saturation intensity of the dye, and the resulting fluorescence enhancement is governed by the modified radiative decay rate of the fluorophore. In this case, the enhancement is largest if the fluorescence emission maximum coincides with the LSPR. In other words, our approach exploits PSF deformations to localize the fluorophore on the particle surface and thereby reveals that imagers binding to the tip of the nanocone are excited above the saturation intensity of the dye, but imagers binding to the base remain in the weak excitation regime. When switching the excitation to s‐polarization with predominant excitation of the base resonance, no such trends in either the mean or maximum EF are visible (Figure 5c,d). The fluorescence enhancement at the base, which is excited in both polarizations, is similar under p‐ and s‐polarization for the larger nanocones but increases for p‐polarization toward smaller nanocones, which might indicate that the enhanced near‐field volumes around the tip and base start to overlap.

A small deviation in collection efficiency for base versus tip binding is furthermore expected due to the different radiation patterns for either case, leading to a potential underestimation of the tip EF. This deviation depends on the numerical aperture (NA) of the used objective lens and is deemed negligible for high‐NA lenses, as chosen here (NA = 1.49).

To further refine the vertical localization of each event, finding the best fit of its corresponding PSF to numerical calculations is introduced in a second approach (see Section 4 and Figures S7–S9 for details). This allows for a binding‐height classification not only for donut‐shaped PSFs but also for Gaussian shapes stemming from the base. The results are used to gain further insights into the binding‐position dependence of the fluorescence enhancement factor around the nanocones.

**FIGURE 6:**
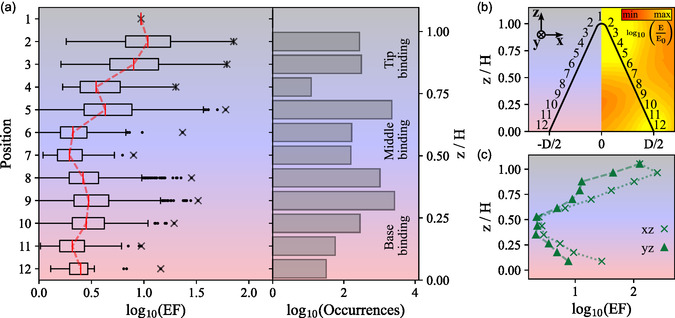
(a) Binding‐height related fluorescence intensity EF and absolute occurrences per 10 min of binding events to ∼50 CR nanocones with *H* = 116 nm under p‐polarized excitation (*z*/*H* denotes the normalized binding height, quantities are shown in logarithmic scaling for clarity). The binding position is obtained by finding the best fit to simulated PSFs for 12 positions along the nanocone shell as indicated in (b). Red markers correspond to the median enhancement factor for each position (analogous figures for all measured particle sizes are found in Figures S10 and S11). (b) Schematic representation of binding positions used for height correlation (left) and simulated electric near‐field enhancement around the nanocone (right, logarithmic scaling). (c) Simulation of the height‐dependent fluorescence intensity enhancement factor at a nanocone with equal dimensions (see Supporting Information part B for details on the enhancement factor calculation). The evaluation is done along the nanocone surface in the *xz*‐ and *yz*‐plane, respectively, with the total internal reflection excitation propagating in the positive *x*‐direction.

In Figure 6a we show the results for p‐polarized excitation and nanocones with a height of *H* = 116 nm (see Figures S10 and S11 for all nanocone sizes and for s‐polarization). The median enhancement factor follows a decreasing trend from the tip toward a minimum in the middle of the nanocone (positions 6,7), whereas the maximum fluorescence enhancement is not observed directly above the tip but instead slightly below. This is due to the contribution of radiative rate enhancement as well as excitation enhancement, where the latter can be higher slightly below the tip depending on the azimuthal position on the nanocone, in line with numerical simulations (see Figure 6b,c and S12b). For lower binding heights, an increase of the enhancement factor is visible again, corresponding to the influence of the base mode electric field enhancement. The highest numbers of detected binding events are found in the upper middle section of the nanocones as well as the upper base region (see Figure 6a right panel), suggesting a higher probability of binding to these areas. Direct binding to the tip as well as to the low base region is more unlikely due to either the small available surface area for the first case, or steric obstruction by the substrate for the latter. Altogether, the detected frequency at different binding heights is also influenced by the homogeneity of functionalization using the docking strand and thus may vary between different particles. It should be noted that fabrication artifacts, such as skewness of the nanocone, can lead to a larger uncertainty in the binding position determination. Furthermore, the accuracy of the binding height determination ultimately depends on the resolution of the recorded PSFs as well as the duration of individual binding events (which determines the total number of collected photons). We estimate the accuracy to be in the order of ±10 nm, with even better localization in the middle section (cf. Figure S9). This approach of position localization by comparing experimental to known PSFs would be well suited for automatization and machine learning classification algorithms [47, 48].

### 2.5 Location‐Dependence of Single‐Molecule Spectral Reshaping

In addition to the intensity enhancement, also the location‐dependent spectral shape of the emitted fluorescence is investigated by collecting the first diffraction order of the emitted signal. The obtained spectra of each single‐molecule binding event are deconvoluted (see Supporting Information part A) and correlated with the shape of the respective PSF being imaged in the zeroth order to distinguish between tip, middle, and base binding.

**FIGURE 7:**
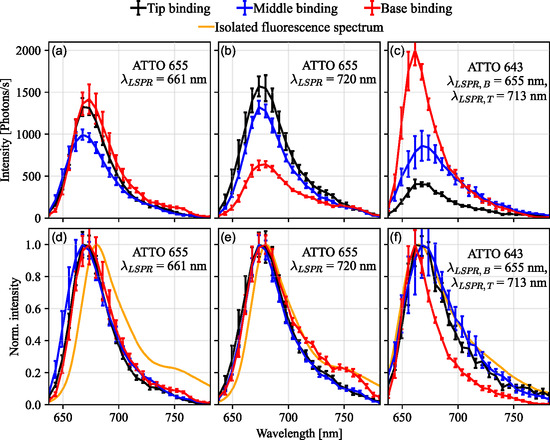
(a) Averaged fluorescence spectra of tip, middle and base binding events of ATTO 655 fluorophores to CR nanocones with a resonance wavelength of 661 nm. Spectra with the 10 highest intensities for each nanocone geometry were chosen for averaging, and the error bars correspond to the standard error of the sample mean. (b) Same as (a) but for CR nanocones with resonance wavelengths of 720 nm. (c) Averaged fluorescence spectra of tip, middle and base binding events of ATTO 643 to SR nanocones with base resonances of 655 nm and tip resonances of 713 nm. (d–f) Same as above with normalized fluorescence signal and comparison to the respective isolated fluorophore spectra. Complementary simulations on the spectral fluorescence reshaping are found in Figures S6 and S12. All spectra were taken using p‐polarization.

Averaged fluorescence spectra of the three types of binding events for three different nanocone geometries are shown in Figure 7. The spectra are fitted with an exponentially modified Gaussian function (see Supporting Information part A) to obtain the peak maxima and FWHMs. ATTO 655 fluorophores are bound to CR nanocones with spectrally inseparable plasmonic tip and base resonances ($\lambda_{\text{LSPR}}$ = 661 nm for Figure 7a,d, $\lambda_{\text{LSPR}}$ = 720 nm for Figure 7b,e), while ATTO 643 molecules are bound to SR nanocones with spectrally separable tip ($\lambda_{\text{LSPR},T}$ = 713 nm) and base ($\lambda_{\text{LSPR},B}$ = 655 nm) resonances (Figure 7c,f). For the smallest CR nanocones with $\lambda_{\text{LSPR}}$ = 661 nm (Figure 7a,d), no significant spectral differences between tip, middle, and base binding are observable. However, compared to the isolated fluorophore emission spectrum, a blue shift of the emission maximum of ∼10 nm is observed for all binding positions. This is explained by the short LSPR wavelength of the nanocones, which enhances the main fluorescence transitions of ATTO 655 more efficiently, while the vibronic tail appears reduced. This changes for CR nanocones with $\lambda_{\text{LSPR}}$ = 720 nm (Figure 7b,e), where the fluorescence spectra stemming from binding to the base resemble the isolated fluorescence spectrum more closely, while vibronic transitions in spectra from tip and middle binding are still relatively reduced. This indicates a larger overlap of the base resonance with the vibronic tail and thus a stronger spectral enhancement for larger wavelengths. The implicit spectral difference between tip and base mode is, however, not observable in the corresponding scattering spectra due to the large FWHM of the resonances.

It should be noted that higher‐order modes could have an influence on the fluorescence signal as well [49]. In the case of the nanocones used in this work, such a quadrupolar mode is present, however, deemed to be of negligible influence, as it is far blue‐shifted compared to the relevant fluorescence transitions.

More pronounced effects are observable for nanocones with spectrally separable tip and base resonances (Figure 7c,f). The intensity for base binding is more strongly enhanced due to the higher overlap between the plasmonic base mode and main fluorescence transition compared to the plasmonic tip mode, which falls into the vibronic tail. Here, the spectral shape between base and tip‐/middle binding differs significantly, most prominently in the reduction of the linewidth for base binding events, yielding a FWHM of ∼34 nm compared to tip and middle binding (47–56 nm). The observed spectral differences are thus ascribed to the spectral position of the LSPRs relative to the isolated fluorophore emission spectrum as is well known from literature. They are in good qualitative agreement with corresponding simulations (see Figure S6b and S12c,d). Thus, a correlation of the spectral shape to the location of the emitter relative to the particle is possible taking into consideration the plasmonic resonances of the tip and base mode. In essence, the position localization serves to analyze the height‐dependent coupling to each plasmonic mode and ultimately the local density of optical states.

## 3 Conclusion

We demonstrate a multimodal quantification of the interaction of isolated plasmonic nanocone antennas with individual, transiently bound fluorophore molecules in a hybrid nanoparticle‐nanoemitter system. Location‐specific PSF deformations are exploited for an estimation of the binding height on the nanocone surface in order to correlate the position to spectral features of the hybrid system. We experimentally demonstrate that the binding position dictates the spectral shape as well as the intensity of the emitted fluorescence as a result of variable coupling to orthogonal nanocone resonances. This spectral information is put into context with the plasmonic antenna properties obtained by experimental and numerical techniques. The method yields high statistics by the parallel measurement of large numbers of particles, while avoiding the need for manual fluorophore positioning. From these statistics, an antenna‐size dependence of the detection rates is identified. Compared to the purely geometrical surface area (64% base and 36% tip contribution), more strongly preferred base binding is observed for smaller nanocones, whereas binding to the upper section is increasingly favored for larger nanocones. This is understood by the larger binding affinity to high‐curvature areas of the particle, size‐dependent modification of the fluorescence emission, as well as height‐dependent diffusion constants over the interface. Statistical evaluation shows that the fluorescence intensity enhancement is largest at or just below the nanocone tip, yielding up to 110× enhancement when using particles with suitable resonances. For base binding, the highest enhancements are found to be ∼40×. The observed trends are in good agreement with numerical expectations on the location‐dependent fluorescence enhancement. The size‐dependent fluorescence enhancement furthermore indicates that the fluorophores may be excited into different regimes depending on the binding position on the particle and can exceed saturation in the case of tip binding. In addition, trends in the spectral shape are in line with theoretical predictions on site‐specific spectral reshaping.

Altogether, the presented method of vertical emitter localization by PSF deformation analysis enables the high‐resolution large‐scale correlative study of location‐dependent decay rates and emission characteristics. It is generally suited for particles that offer at least one plasmonic resonance with out‐of‐plane orientation. The use of nanocones has the added advantage that small volumes with high near‐field enhancement can be probed near the nanocone tip, and that different excitation regimes and influences of substrate effects may be investigated on the same particle. The approach thus promises unique access to otherwise inaccessible information, such as the modification of diffusion properties and binding kinetics near liquid‐solid interfaces. The method could be expanded to azimuthal binding‐position evaluation. Likewise, temperature‐dependent binding kinetics in DNA‐hybridization assays or the modification of binding statistics under different flow velocities in microfluidics may be investigated. By extension to further emitter species or multiplexed sensing, insights into preferred binding sites due to steric effects may be gained.

## 4 Methods

### 4.1 Fabrication of Gold Nanocones

Fabrication of nanocones can be achieved by means of electron beam lithography, etch‐mask transfer, nanoimprint lithography, or others [6, 50, 51, 52, 53]. For this study, gold nanocones with different geometries are fabricated on glass substrates following a previously described etch‐mask transfer method [54, 55]. Glass cover slips (Menzel, thickness 170 µm) are sputter coated (Univex 300 sputter coater) with a 50 nm layer of indium tin oxide (ITO) for providing adhesion between the gold particles and the glass as well as providing electrical conductivity for SEM investigation. A gold layer is then deposited using thermal evaporation (Balzers BA510), the thickness of which determines the maximum height of the subsequently formed gold nanocones. For nano‐patterning, a 120 nm thick polymethylmethacrylate (PMMA) layer is spin‐coated (Süss MicroTec Delta 6 RC). Using electron beam lithography (FEI XL‐30 FEG equipped with Xenos pattern generator), multiple 100 × 100 µm^2^ fields with 25 × 25 point exposures are written with increasing dose. The exposed points are separated 4 µm from each other to later avoid optical coupling between the individual nanoparticles. Development is then performed in a 3:1 isopropanol–methylisobutylketone mixture, and the sample is treated with a mild oxygen plasma to remove PMMA residues in the cavities. A layer of aluminum oxide is evaporated afterwards using electron beam evaporation (Balzers BA510). After lift‐off, the remaining aluminum oxide discs act as etch‐masks in a subsequent argon ion milling step (Roth & Rau Unilab) in which the conical shape is formed. Here, the etching parameters may be tuned to produce nanocones with varying aspect ratio, and the sample holder is rotated steadily during milling. After sufficient etching with a suitable choice of the thickness of the aluminum oxide layer, gold nanocones with tip radii of 5–10 nm are formed. An overview of the fabricated nanocone sizes is found in Table S1.

### 4.2 Determination of Localized Surface Plasmon Resonances Using Dark‐Field Spectroscopy

An inverted Nikon Eclipse Ti‐S microscope equipped with a 60x Nikon S Plan Fluor (NA = 0.7) air objective is used for the acquisition of spectra. Unpolarized white‐light illumination is provided by a halogen lamp and is focused on the sample using a Nikon dark‐field condenser (NA 0.95–0.8). The microscope is coupled to an Andor SR‐303i‐B spectrograph equipped with an Andor iDus 416 CCD camera. A 150 lines/mm grating is used, resulting in a scan range of 400–1000 nm. The scattering spectra are corrected by the background signal of the sample and normalized to the lamp spectrum minus the dark current of the camera. To ensure that the refractive index matches that of the medium in the fluorescence studies, the nanoparticles are immersed in purified water (Milli‐q 18.2 MΩ cm).

### 4.3 Nanoparticle Functionalization by DNA‐Mediated Fluorophore Binding

A sample chamber is constructed by sticking a silicone isolator (Grace Bio‐Labs) with a central aperture of diameter 8 mm to the sample substrate. This chamber is filled with a solution of 5 μM docking strands (Integrated DNA Technologies; see Table 1 for DNA sequences) and 1 mM tris(2‐carboxyethyl)phosphine hydrochloride (TCEP, Merck) in citrate buffer (10 mM, pH 3, 1 M NaCl). After 2 h incubation time, the sample is rinsed with PBS followed by buffer B (5 mM Tris‐HCl, 10 mM MgCl_2_, 1 mM EDTA, pH 8.0, filtered) to remove excess docking strands and stored in the fridge for a day before use. The imaging solution (1 nM imager strands (Eurofins Genomics) in buffer B) is then pipetted onto the sample, and the chamber is covered with a cleaned glass slide to prevent evaporation.

**TABLE 1:** DNA sequences of docking and imaging strand oligonucleotides.

Strand name	Sequence
Docking strand	5′‐thiol‐CAT CAT CAT ACG CTT CCA ATA *ATA CAT CTA*‐3′
Imager strand	5′‐C *TAG ATG TAT*‐ATTO 655/643‐3′

### 4.4 Microscopy Setup for Fluorescence Emission Characterization

The sample is placed on an inverted wide‐field microscope (Nikon Ti2) and illuminated by a 637 nm excitation laser (OBIS FP 637 LX, Coherent) through a 100x oil‐immersion objective (NA 1.49) in total internal reflection mode, with an excitation intensity of ≈2 × 10^7^ W/m^2^ (cf. Figure 1a). A lambda‐half waveplate is placed in the excitation path to select either s‐ or p‐polarization. A dichroic mirror (ZT640rdc, Chroma), a notch filter (ZET635NF, Chroma), and a long‐pass filter (FELH0650, Thorlabs) are placed in the detection path to remove the reflected laser light. The signal is captured on an EMCCD camera (Andor iXon Ultra DU‐888). For acquisition of the spectral information, a 70 lines/mm grating (Edmund Optics) is introduced before the camera.

### 4.5 Numerical Simulation of Plasmonic Resonances

For an estimation of the desired nanoparticle dimensions, numerical simulations using the FEM implemented in Comsol Multiphysics v6.2 are conducted prior to particle fabrication. Simulation geometries are modeled to mimic the corresponding experiment setups closely. Scattered radiation excited under dark‐field illumination is collected on a spherical segment that corresponds to the numerical aperture of the objective (NA = 0.7), and full wavelength‐dependent refractive indices are used for the particle and substrate. Refractive indices for a polycrystalline gold film with a thickness of 117 nm are used [56]. The 50 nm thick ITO‐layer is modeled with data obtained by König et al*.* for a 72 nm thick film [57], and glass is modeled with *n* = 1.5. The medium is chosen to be water with refractive index *n* = 1.33. For the later performed reshaping simulation, particle geometries are chosen to match the experimental scattering spectra. Tip radii are defined as measured by SEM (see Table S1), and the radius of curvature at the base is set to 1 nm. Details on complementary fluorescence enhancement and spectral reshaping simulations are found in the Supporting Information part B.

### 4.6 Numerical Simulation of PSF Shape and PSF Classification

#### 4.6.1 Numerical PSF Calculation

To calculate the PSFs for emitters at different positions on the gold nanocone, we follow the procedure by Huijben et al. [33]. In short, the numerical approach is divided into three parts. First, we use finite‐difference time‐domain simulations (FDTD, Lumerical Inc., 3D Electromagnetic Simulator, version 2021, release 1.3) to calculate the electromagnetic field in the top part of the sample, up until just below the ITO layer where the near field is detected. The simulation domain is 8 µm × 8 µm × 300 nm, with a mesh accuracy parameter of 6, a locally refined mesh size around the nanocone of 3 nm, and a simulation time of 40 fs. The ITO layer is 50 nm thick (*n* is approximated to 1.74) and the glass layer (*n* = 1.52) stretches from below the ITO layer until the bottom of the FDTD simulation domain. The near‐field detector is located 20 nm below the ITO‐glass interface. The gold nanocone (diameter = 100 nm, height = 104 nm, tip radius = 0 nm, *n* of gold according to ref. [58]) is positioned on top of the ITO layer. Secondly, the detected near field is propagated into the far field using the Lumerical built‐in function *farfieldexact* onto a hemisphere with a radius of 1 m. The calculated far field is focused onto the camera by using the Debye‐Wolf diffraction integral, implemented in Matlab.

The fluorophore is modeled as a freely rotating dipole, emitting at 675 nm. The PSF of a freely rotating dipole emitter is obtained by averaging the PSFs for three orthogonal dipole orientations (*x*‐, *y*, and *z*‐direction) [33, 59]. We calculate the PSFs for multiple fluorophore positions along the nanocone separated 5 nm from the gold surface, equally spaced between the tip and the base of the nanocone (see Figure 3a,d and S3).

For mapping the position‐dependent fluorescence enhancement factor to corresponding PSFs, the setup is modeled using Comsol Multiphysics v6.2. The same dimensions of the nanocone as well as the material properties are used as above, and the near fields are evaluated on a 1 µm × 1 µm plane located 20 nm below the ITO‐glass interface. Then, the detected near fields are analytically propagated into the far field using the angular spectrum representation. As above, the fluorophore is modeled by three orthogonal dipoles emitting at 675 nm, and the PSFs are calculated for 12 dipole positions along the nanocone at 5 nm distance from the gold surface.

#### 4.6.2 PSF Classification

We classify the PSF shapes into groups representing the binding location on the nanocone by differentiating between donut‐ and elliptical Gaussian‐shapes. We perform the classification as follows. First, fitting a 2D Gaussian using maximum likelihood estimation [38] gives us an estimate for the center of the PSF. Secondly, we interpolate the intensity on circular trajectories for multiple radii centered around the PSF center. For each circular trajectory, we compute the average intensity, where we take the radius with the largest average intensity as the optimal radius of the PSF shape. For donut‐shaped PSFs, the found radius represents the radius of the donut, while for elliptically‐shaped PSFs, the radius will be zero. We classify a PSF as a donut (tip‐ or middle‐binding) when the radius is larger than 1 pixel and as an ellipse (base‐binding) when the radius is smaller than 1 pixel.

We can further classify the donut PSFs into symmetric (binding at the tip of the nanocone) and asymmetric (binding in the middle section of the nanocone) by investigating the intensity profile at the previously determined radius around the center of the PSF. From the azimuthal intensity profile, we find the maximum and minimum values and compute the ratio $R=\frac{I_{\max}-I_{\min}}{I_{\min}}$. A small (near‐zero) ratio represents a very symmetric donut (binding at the tip), and a larger ratio represents a more asymmetric donut (binding in the middle). We classify a PSF as tip‐binding when the ratio is smaller than 0.3, and as middle‐binding when the ratio is bigger than 0.3.

For the refined PSF mapping, squared residuals are calculated between each pixel of the experimental and simulated PSFs, respectively, and summed up. This is repeated for all simulated positions and orientations in order to find the best fit (see also Figures S7–S9 and part C for an estimation on the accuracy of the position determination).

### 4.7 Statistical Analysis

Particle sizes are given as mean ± standard deviation, where 3–6 particles per size are measured using SEM. Dark‐field scattering spectra are taken on 6 different particles per fabricated size and are averaged. The LSPR wavelengths as well as FWHMs are evaluated as mean ± standard deviation, obtained by single‐ or double Lorentzian curve fitting.

For fluorescence EFs, binding events at 40–60 nanocones are monitored in parallel over a period of 10 min per fabricated size and used polarization. Weak signals are excluded from the dataset by thresholding, resulting in ∼155 events per nanoparticle. Events at all 40–60 nanocones per measured size and polarization are collectively evaluated in one dataset respectively. The fluorescence intensity enhancement is given as mean ± standard error of the mean and is calculated by subtracting the luminescence of the nanocones when no binding event occurs. The result is then divided by the averaged non‐enhanced fluorescence intensity of molecules diffusing to the glass‐water interface.

For spectral reshaping, binding events of 12 nanocones per measured size are monitored and correlated to the respective spectra. The 10 spectra with the highest intensity are averaged for each binding location, and the spectra are given as mean ± standard error of the mean.

Processing of data as well as visualization is carried out using Python andMatlab.

## Supporting Information

Additional supporting information can be found online in the Supporting Information section.

## Conflicts of Interest

The authors declare no conflicts of interest.

## Supporting information

Supplementary Material

## Data Availability

The data that support the findings of this study are available from the corresponding author upon reasonable request.
