# Supplementary material for: Single‐Molecule Imaging and Spectroscopy Enables Quantification of Location‐Dependent Light–Matter Interactions on Nanoantennas
Source: Small Sci. 2026 Mar 5;6(3):e202500597. doi: 10.1002/smsc.202500597 (PMC12970204; doi:10.1002/smsc.202500597)
Supplement: Supplementary file 1 — Supplementary Material [file SMSC-6-e202500597-s001.pdf]

Supplementary Information

Single-molecule Imaging and Spectroscopy Enables Quantification of Location-dependent Light-matter Interactions on Nanoantennas

Lukas Lang, Sjoerd Nooteboom, Teun A. P. M. Huijben, Sarojini Mahajan, Rodolphe Marie, Peter Zijlstra*, Monika Fleischer**

L. Lang*, M. Fleischer*
Institute for Applied Physics and Center LISA[+], Eberhard Karls University Tübingen, 72076 Tübingen, Germany
E-Mail: lukas.lang@uni-tuebingen.de, monika.fleischer@uni-tuebingen.de

S. Nooteboom, S. Mahajan, P. Zijlstra*
Department of Applied Physics and Science Education, Eindhoven University of Technology, 5600 MB Eindhoven, The Netherlands
E-Mail: p.zijlstra@tue.nl

T.A.P.M. Huijben, R. Marie
Department of Health Technology, Technical University of Denmark, 2800 Lyngby, Denmark

Keywords: single-molecule spectroscopy, plasmonics, nanofabrication, localization microscopy, nanoantenna, single-molecule imaging, nanophotonics, point-spread function

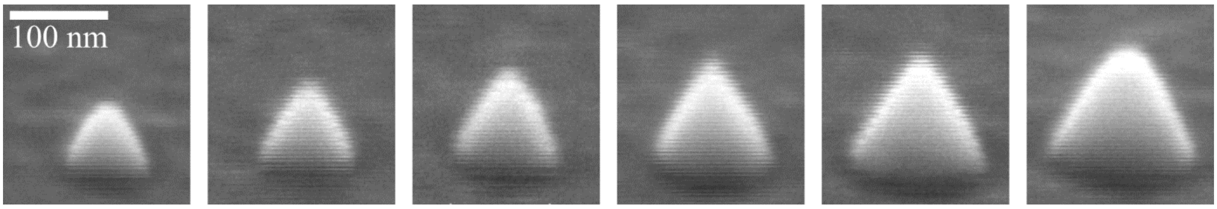

Fig. S1: SEM images of fabricated CR gold nanocones for all investigated sizes. The viewing angle is 60°. The particle heights H are from left to right: 89 nm ± 7 nm, 93 nm ± 2 nm, 104 nm ± 5 nm, 116 nm ± 3 nm, 123 nm ± 2 nm, 125 nm ± 7 nm.

Table S1: Averaged particle geometries of investigated nanocones as determined by scanning electron microscopy (SEM). Averaging is done over 3 to 6 particles per size. CR: spectrally coinciding tip and base resonance, SR: spectrally resolved resonances.

	λ_{LSPR} [nm]	Height H [nm]	Diameter D [nm]	Tip diameter [nm]
CR	738 ± 4	125 ± 7	131 ± 3	28 ± 1
	720 ± 3	123 ± 2	116 ± 3	15 ± 1
	691 ± 7	116 ± 3	106 ± 6	11 ± 1
	676 ± 4	104 ± 5	99 ± 2	11 ± 3
	661 ± 6	93 ± 2	89 ± 3	13 ± 2
	655 ± 3	89 ± 7	76 ± 2	13 ± 2
SR	655 ± 4, 713 ± 10	110 ± 12	99 ± 6	22 ± 3

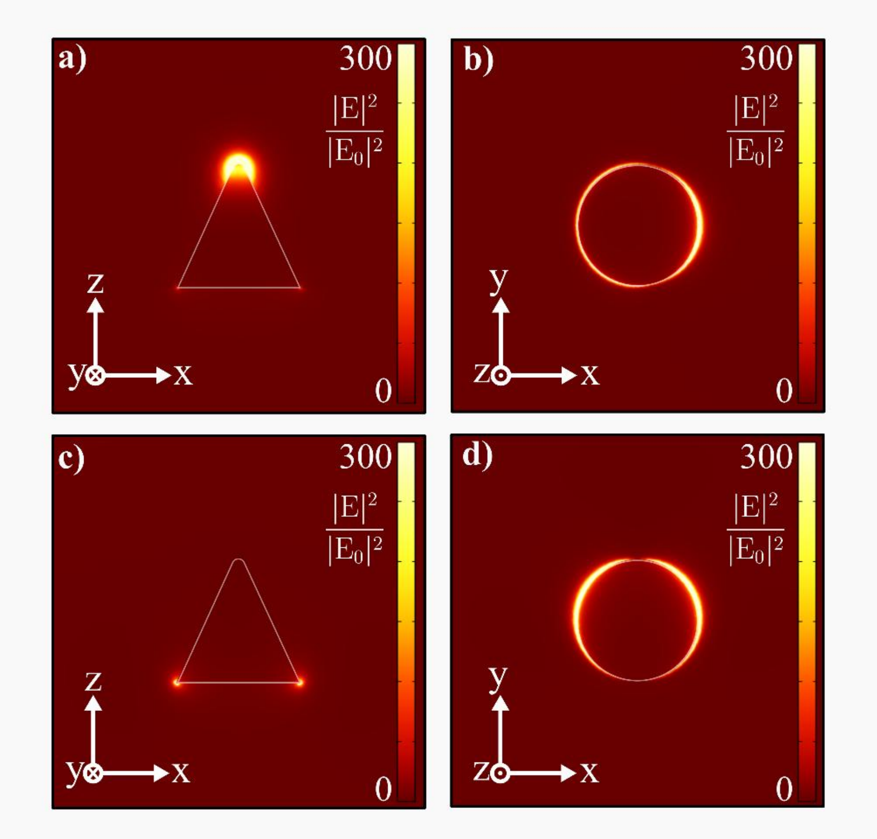

Fig. S2: a) Vertical and b) horizontal cross section of finite element method (FEM) simulations of the electric intensity enhancement around a nanocone on ITO/glass immersed in water using p-polarized excitation (in b) and d) the cross section is evaluated at the substrate interface). Strong enhancement is achieved at the cone tip and the base. c), d) same as a), b) but for s-polarized excitation. The intensity is strongly enhanced predominantly at the base.

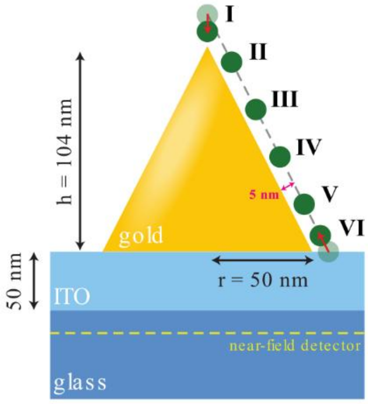

Fig. S3: Details regarding the simulation geometry and fluorophore positions for numerical point-spread function (PSF) calculations using FDTD and FEM calculations. The modeled system consists of a gold nanocone (radius = 50 nm, height = 104 nm) on top of a 50 nm ITO layer on a glass layer. The electromagnetic field is detected at a near-field detector (yellow dotted line) placed 20 nm below the ITO-glass interface. For the PSF calculations, we consider multiple fluorophore positions (green dots) separated 5 nm from the gold surface (grey dotted line), equally spaced between the tip and the base of the nanocone. Position 1 is lowered to be 5 nm above the tip of the cone, and the lowest position is moved along the grey dashed line to be 5 nm from the ITO surface. For clarity only six positions are indicated in the Figure

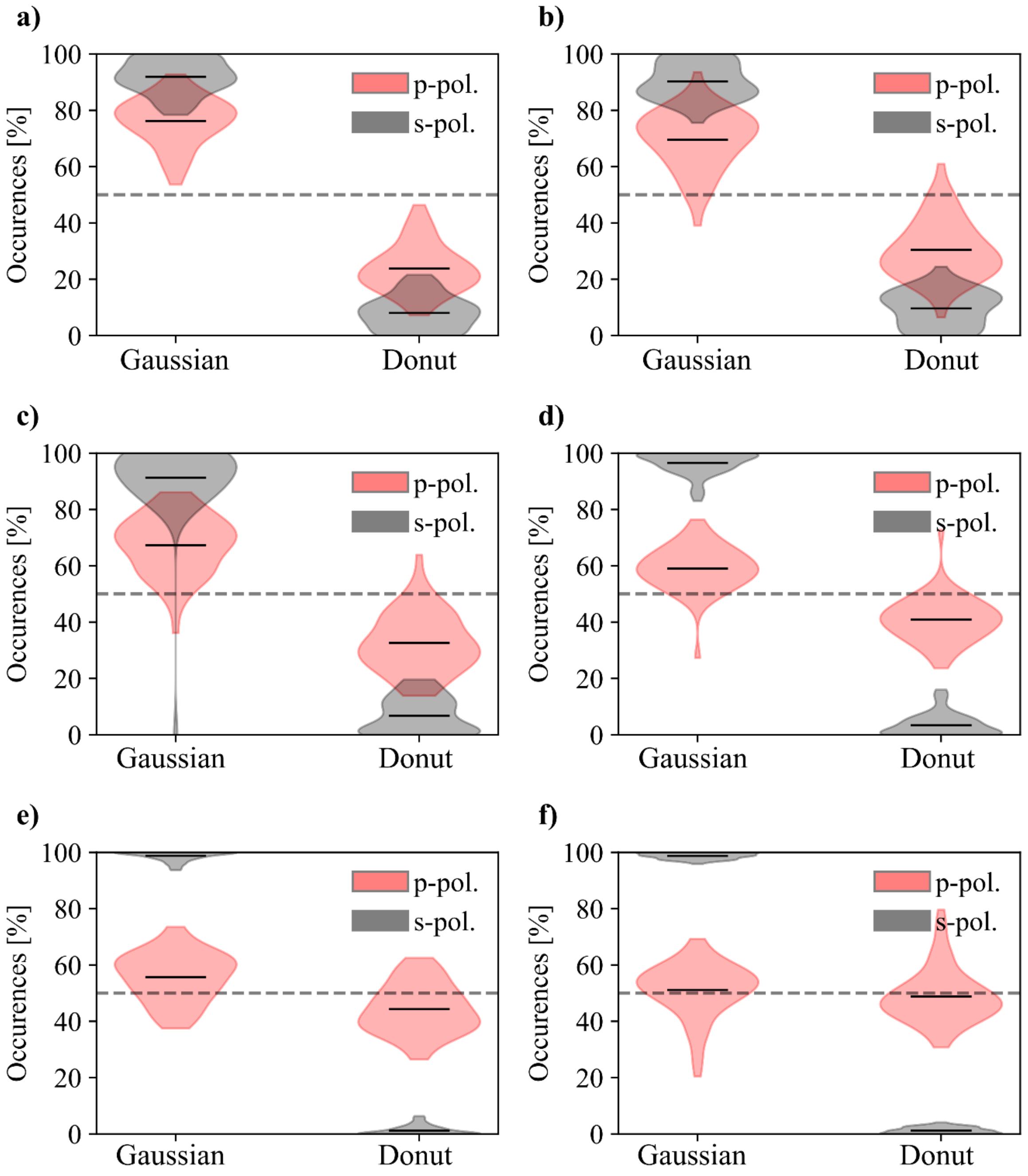

Fig. S4: Occurrences of detected donut- and Gaussian-shaped PSF events in percent of the total number of detected events on CR nanocones with increasing size, for p-polarized and s-polarized excitation. The particle heights are 89 nm ± 7 nm (a), 93 nm ± 2 nm (b), 104 nm ± 5 nm (c), 116 nm ± 3 nm (d), 123 nm ± 2 nm (e), 125 nm ± 7 nm (f).

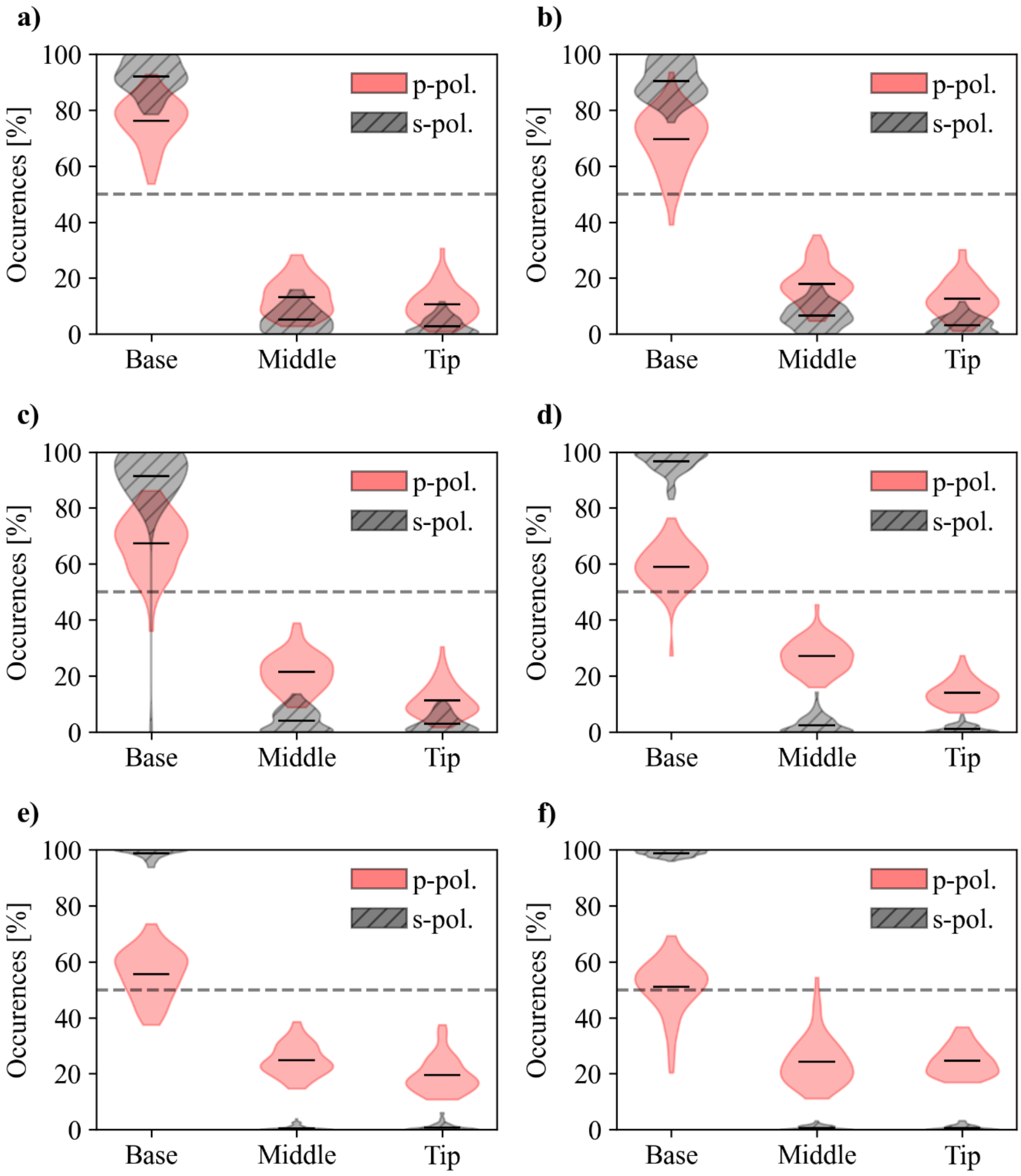

Fig. S5: Occurrences of detected tip-, middle- and base-binding events in percent of the total number of detected events on CR nanocones with increasing size, for p-polarized and s-polarized excitation. PSFs with an asymmetry ratio < 0.3 are attributed to binding to the tip, while PSFs with ratio ≥ 0.3 are attributed to the middle section of the cone. Gaussian-shaped PSFs are attributed to the base. The particle heights are 89 nm ± 7 nm (a), 93 nm ± 2 nm (b), 104 nm ± 5 nm (c), 116 nm ± 3 nm (d), 123 nm ± 2 nm (e), 125 nm ± 7 nm (f).

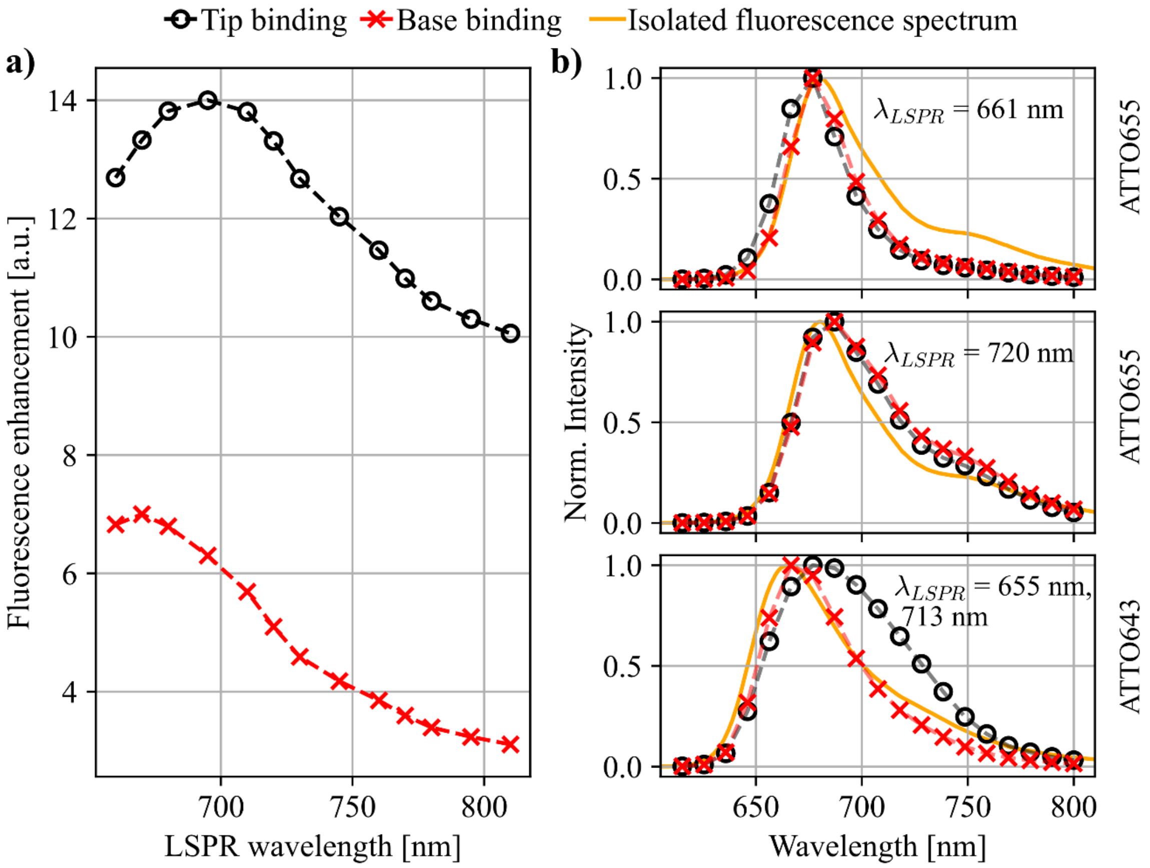

Fig. S6: a) FEM simulation of the fluorescence intensity enhancement evaluated 5 nm above the tip apex and 5 nm besides the base (elevated 5 nm above the substrate) of AR = 1 nanocones with heights ranging from 70 to 130 nm, plotted as a function of the corresponding LSPR wavelength. The intensities are scaled to the maximum value of the measurement (cf. Figure 5a) for comparability. b) Spectral fluorescence reshaping simulations on CR and SR nanoparticle geometries corresponding to the measurement (cf. Figure 7) under p-polarized excitation.

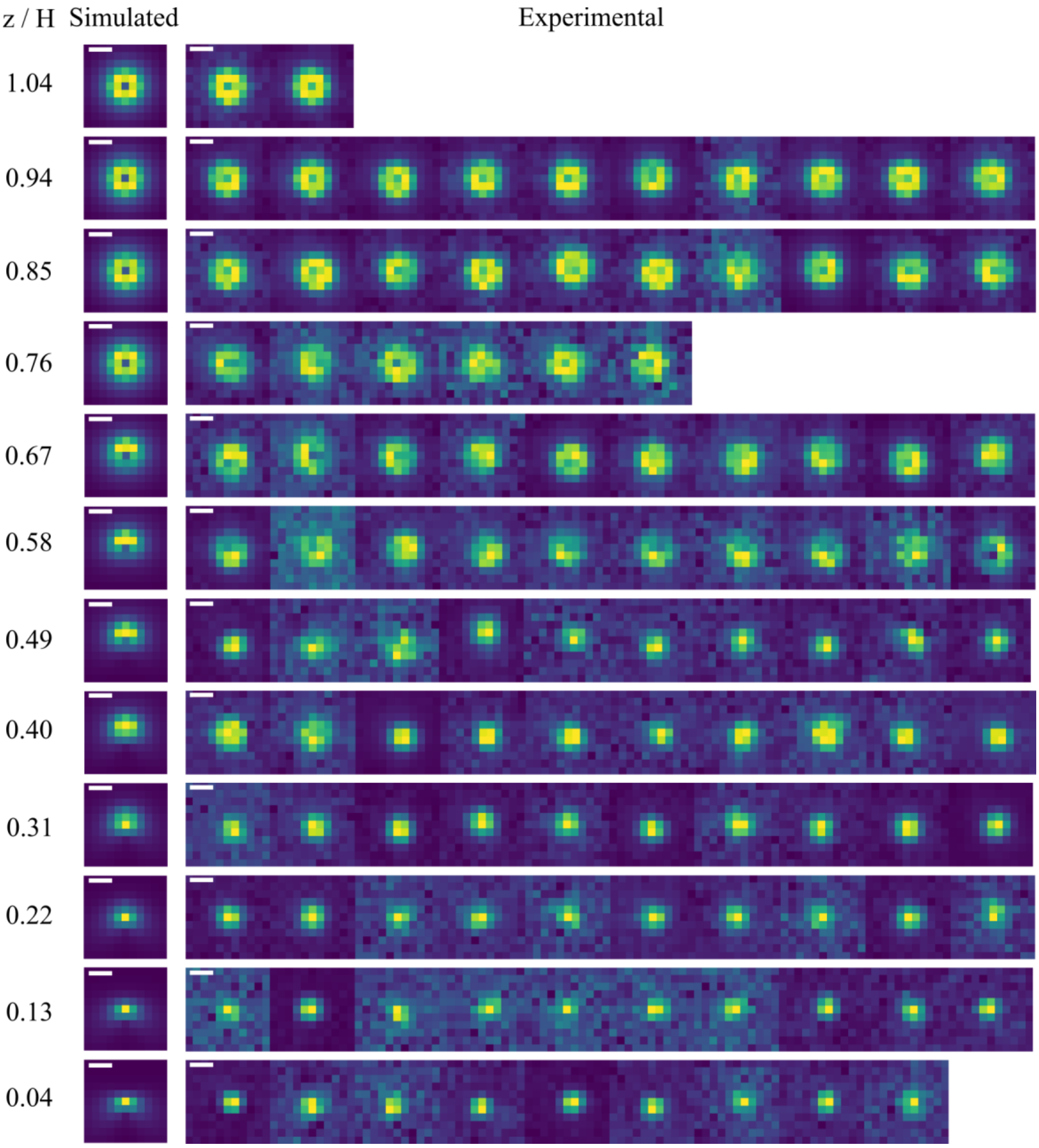

Fig. S7: Collection of exemplary binding-height correlated simulated and experimental PSFs, as a function of the normalized binding height z / H (scale bars are 400 nm). The best fit of the experimental to the simulated PSFs is found by minimizing the summed squared residuals of the region of interest. This is done by testing each experimental PSF versus all 12 simulated PSFs with all azimuthal orientations with steps of 20°. Only binding events with summed squared residuals smaller than the double median of the entire dataset are used for further evaluation. The number of detected events for each position can vary strongly, as is also seen in Figures S10 and S11.

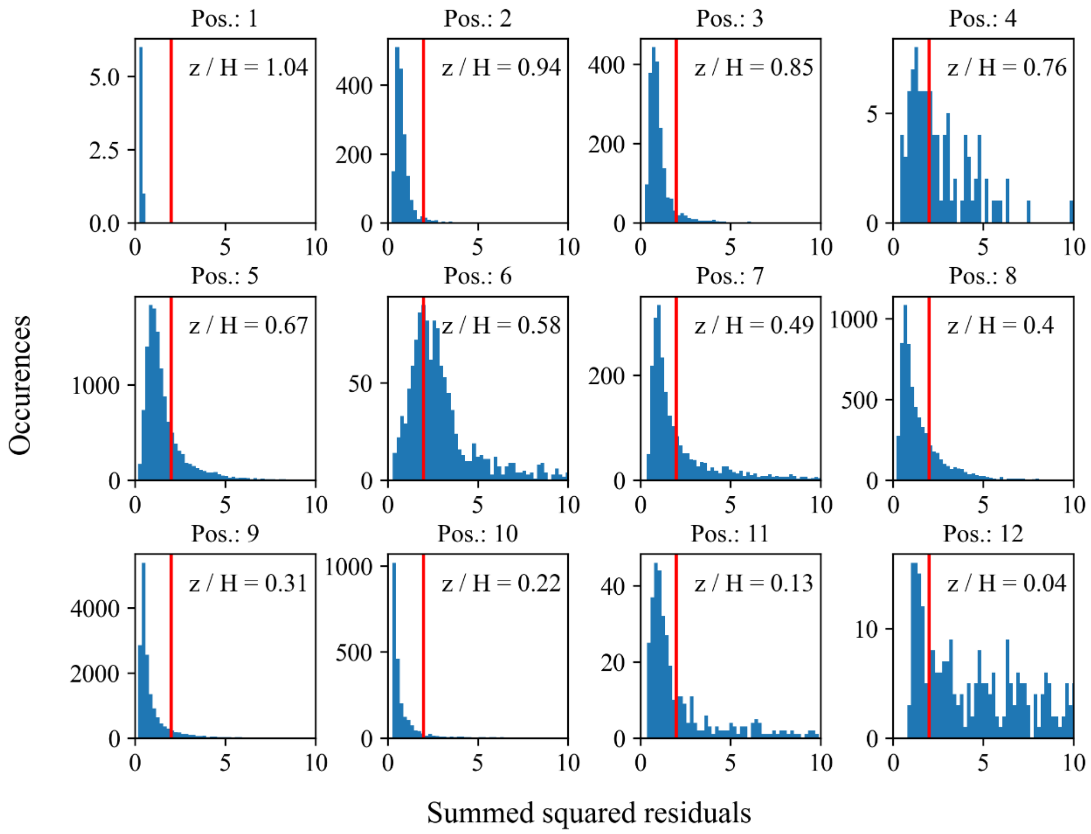

Fig. S8: Minimum summed squared residuals for all events per detected binding position using p-polarized excitation. Red vertical lines denote the double median of ~2 of the entire dataset. Binding events with larger summed squared residuals are discarded for the evaluation. The agreement of the experimental to the simulated PSFs can vary strongly depending on the found position, however large numbers of events fall well below the chosen threshold.

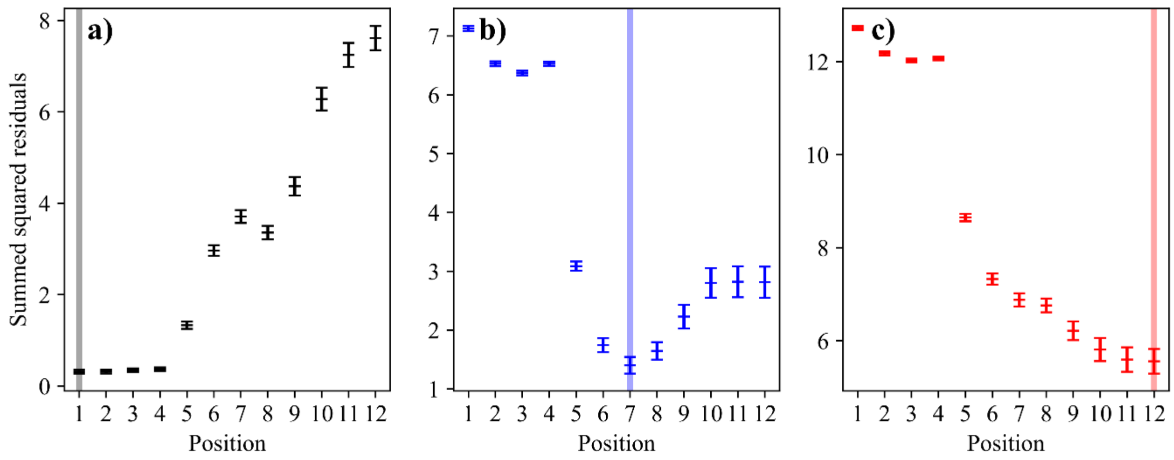

Fig. S9: Summed squared residuals for all simulated PSF positions to experimental ‘position 1’ events a), ‘position 7’ events b) and ‘position 12’ events c). The orientation with the smallest deviation from the experiment was chosen for each position, and the events are correlated to a binding position by finding the minimum of summed squared residuals. Error bars correspond to the standard error of the mean of the entire dataset for p-polarized excitation.

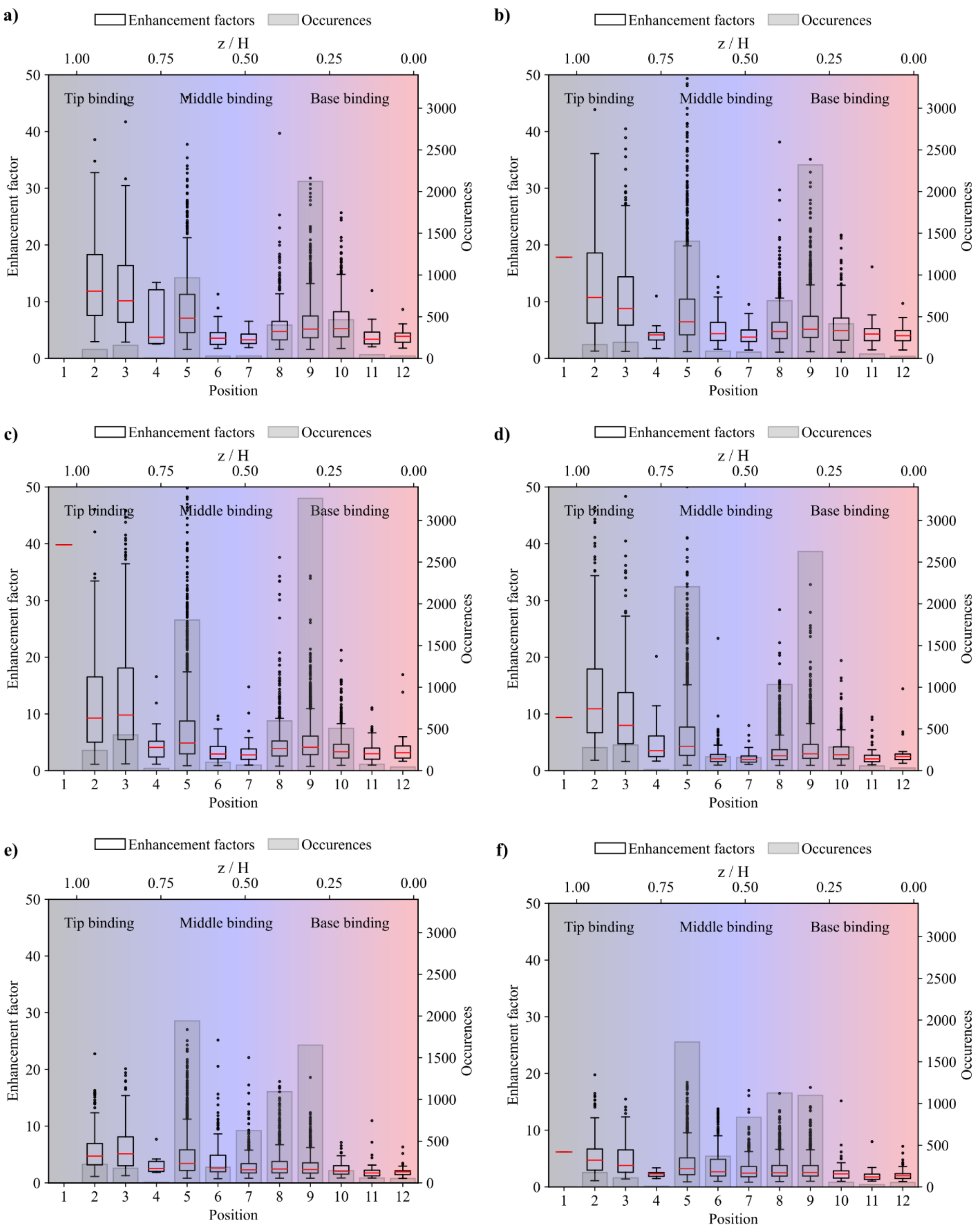

Fig. S10: Experimental fluorescence intensity enhancement factors as well as absolute occurrences over 10 min of binding events to CR nanocones under p-polarized excitation as a function of the binding position and the normalized binding height z / H. The particle heights are 89 nm ± 7 nm (a), 93 nm ± 2 nm (b), 104 nm ± 5 nm (c), 116 nm ± 3 nm (d), 123 nm ± 2 nm (e), 125 nm ± 7 nm (f) (cf. Table S1 for corresponding LSPR wavelengths).

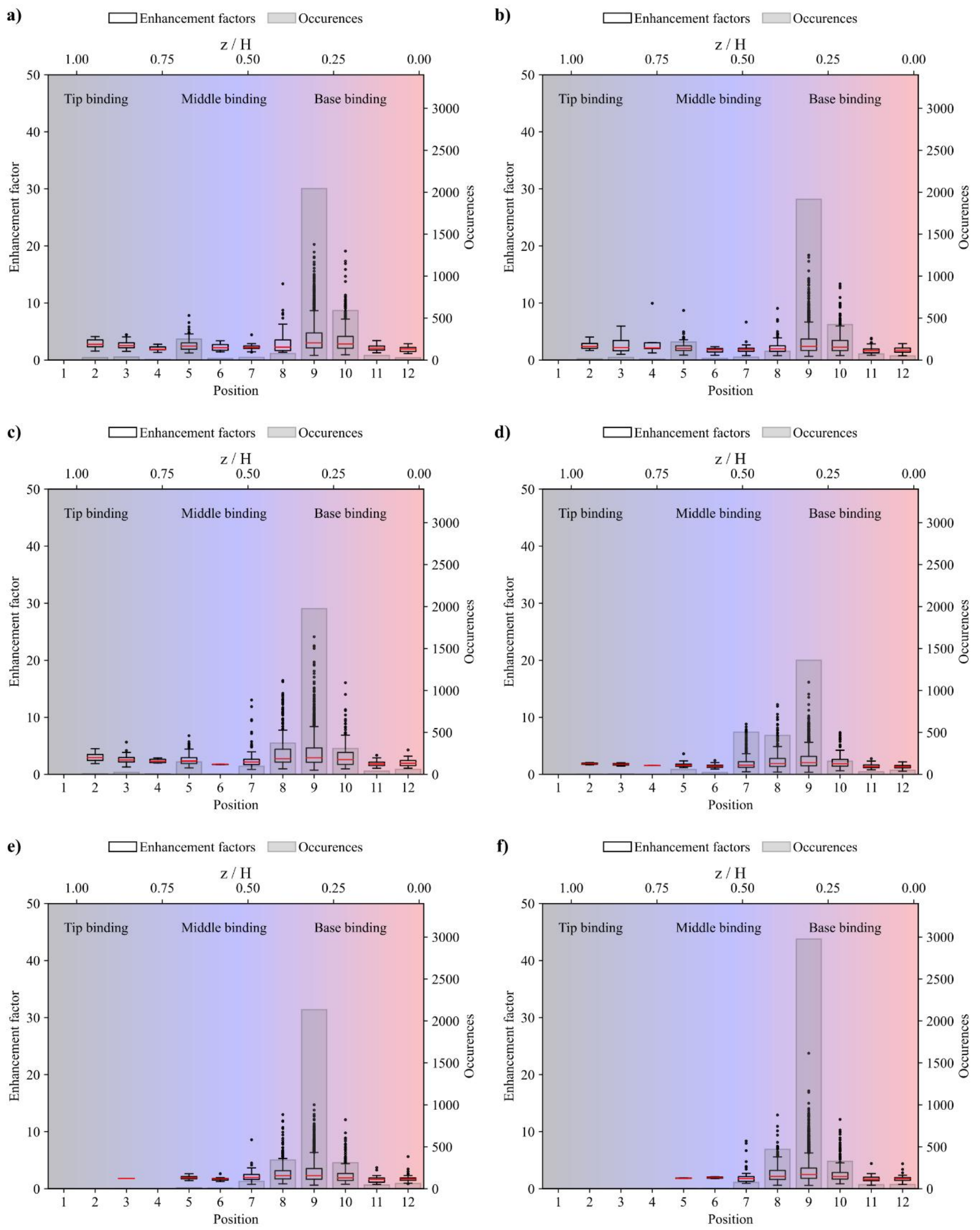

Fig. S11: Experimental fluorescence intensity enhancement factors as well as absolute occurrences over 10 min of binding events to CR nanocones under s-polarized excitation as a function of the binding position and the normalized binding height z / H. The particle heights are 89 nm ± 7 nm (a), 93 nm ± 2 nm (b), 104 nm ± 5 nm (c), 116 nm ± 3 nm (d), 123 nm ± 2 nm (e), 125 nm ± 7 nm (f) (cf. Table S1 for corresponding LSPR wavelengths).

Table S2: Overview on fluorescence intensity enhancement factors (EF) for particles with spectrally coinciding tip and base mode under p-polarized excitation. Mean as well as maximum EFs are shown for tip, middle and base binding as a function of the LSPR wavelength of the corresponding particles.

λ_{LSPR} [nm]	Mean EF – tip binding	Mean EF – middle binding	Mean EF – base binding	Highest EF – tip binding	Highest EF – middle binding	Highest EF – base binding
738 ± 4	5.4	2.9	3.3	20.8	17.4	18.5
720 ± 3	6.5	3.7	3.1	30.0	25.2	27.0
691 ± 7	11.4	5.7	3.8	71.7	60.0	32.8
676 ± 4	13.5	7.2	5.1	113.4	59.5	40.2
661 ± 6	12.2	7.7	6.3	74.5	48.4	40.2
655 ± 3	11.4	8.8	6.4	50.6	56.2	39.7

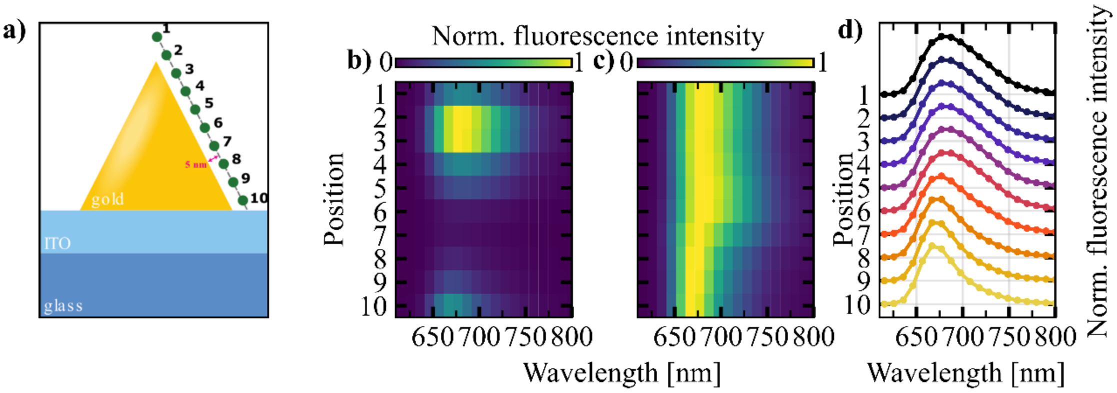

Fig. S12: Spectral fluorescence reshaping simulation on a nanocone with spectrally resolved tip and base mode ($\lambda_{LSPR}^{base} = 655\,nm,\ \lambda_{LSPR}^{tip} = 710\,nm$) with tip radius = 11 nm and ATTO 643 fluorophore binding under p-polarized excitation. a) Overview of the simulated binding positions. b) Position-dependent reshaped fluorescence spectra normalized to the maximum intensity; c) same as b) but with maximum-normalization for each binding position individually. d) Same as c), presented as normalized fluorescence intensity spectra as a function of the wavelength.

A) Numerical analysis of experimental spectral reshaping data

Deconvolution of spectral images by zeroth order PSF:

A larger spatial extension is generally observed for the donut-shaped PSFs upon tip binding compared to Gaussian PSFs for base binding events, leading to artificial broadening of the first order diffraction due to light emanating from a wider range of point sources reaching the grating. The spectral images are deconvoluted by the shape of the PSF using a blind deconvolution algorithm as implemented in MathWorks MatLab v.R2021a. The deconvolution eliminates spectral broadening by the varying spatial extensions of the PSFs.

Curve fitting of obtained fluorescence spectra:

For retrieving peak maxima and peak widths, an exponentially modified Gaussian function is fitted to the experimentally obtained reshaped fluorescence spectra as defined below. In the case of weak spectral reshaping, this function describes the measured spectra well, whereas it has to be used cautiously in the case of strong reshaping with the possible emergence of new peaks.

$$I(x) = \frac{I_0 \nu}{2} e^{\frac{\nu}{2}(2\mu + \nu\sigma^2 - 2x)} erfc\left(\frac{\mu + \nu\sigma^2 - x}{\sqrt{2}\sigma}\right)$$

Here, the mean is defined as $\bar{x} = \mu + 1/\nu$ and the variance as $\sigma_x^2 = \sigma^2 + 1/\lambda^2$, where μ and ν are fitting parameters. $erfc$ denotes the complementary error function.

B) Numerical simulation of fluorescence intensity enhancement and spectral reshaping

Numerical simulations utilizing excitation at the laser wavelength are conducted in Comsol Multiphysics v6.2 to estimate the electric near-field enhancement at the tip and near the base of the gold cones. Using inner reflection with an incidence angle of 70° at the interface between the glass and water half-space separated by 50 nm of ITO, total internal reflection is achieved. The simulation volume consists of a 580 nm x 580 nm x 1300 nm unit cell with periodic boundary conditions along the substrate plane to ensure proper formation of the electric excitation field. For single particle excitation, the periodicity is then lifted, and plasmonic tip and base LSPR modes are excited simultaneously using p-polarization, while only the base mode is excited using s-polarization. The electric field enhancement is evaluated at 5 nm distance from either the tip or the base at the back of the particle in the propagation direction of the evanescent wave in order to mimic the time-averaged distance of the fluorophores to the gold interface.

The fluorescence properties are modeled using electric point dipole excitation over the entire spectral range and averaging over three orthogonal polarization directions to account for fluorophore rotations. The point dipole $\mathbf{p}$ is placed 5 nm above the tip or 5 nm beside the base to compare tip versus base binding. According to the procedure by Ringler et al.,[1] the scattered power of the composite system of nanocone and dipole and the transferred power of the dipole to the cone are collected and normalized by the radiated power of the isolated dipole to obtain enhancement factors for the radiative $g_r(\omega)$ and energy transfer rate $g_{et}(\omega)$. Together with the integral-normalized emission spectra $f_0(\omega)$ of isolated ATTO fluorophores, the spectral probability density for the emission of a photon is evaluated, which is normalized by the quantum efficiency and integral-normalized emission spectrum of the isolated molecule to yield the spectrally resolved enhancement factor for photon emission, $g_{em}(\omega) = \frac{\gamma_{r0} g_r(\omega)}{\eta_0(\gamma_r + \gamma_{et} + \gamma_{nr0})}$. Here, γ_{r0} and γ_{nr0} denote the radiative and non-radiative decay rates of the isolated molecules, and $\gamma_r = \gamma_{r0} \int f_0(\omega) g_r(\omega) d\omega$ and $\gamma_{et} = \gamma_{r0} \int f_0(\omega) g_{et}(\omega) d\omega$ the total emission and energy transfer rates. η_0 is the quantum yield of the isolated fluorophore. The enhanced electric near-field $\mathbf{E}(\mathbf{r_0})$ at the dipole position $\mathbf{r_0}$ under TIR illumination is used to obtain the excitation enhancement

factor at the excitation wavelength $g_{exc}(\omega_{exc}) = \frac{|\mathbf{p}\cdot\mathbf{E}(\mathbf{r_0})|^2}{|\mathbf{p}\cdot\mathbf{E_0}(\mathbf{r_0})|^2}$. Altogether, the product of both enhancement factors and the amplitude-normalized emission spectrum of the isolated dye F_0 yields the reshaped and enhanced fluorescence spectra $F(\omega) = g_{em}(\omega)g_{exc}(\omega_{exc})F_0(\omega)$. Fluorescence intensity enhancement is estimated by integrating the obtained spectra and normalizing by the isolated fluorescence intensity. The particle geometries in the simulations are adapted to conform to the LSPR wavelengths measured during dark-field spectroscopy.

C) Quality of PSF matching and position determination

In order to retrieve the best fit between experimental PSFs and simulated PSFs for each simulated binding height, experimental and simulated data are both normalized to unity. Then the squared residuals of each pixel between the experimental and the simulated PSF are calculated and summed over the entire region of interest (N = 121 pixels per event):

$$summed\ squared\ residuals = \sum_{i=1}^{N}\left(PSF_i^{exp.} - PSF_i^{sim.}\right)^2$$

Per experimental binding event, these sums are evaluated for all simulated positions as well as all azimuthal orientations (steps of 20°), and the minima in the summed squared residuals are extracted to yield the position and orientation. Binding events with summed squared residuals larger than the double median of the entire dataset are discarded for the height-correlated enhancement factor evaluation. The results are summarized in Figure S8, where the distributions of identified minima in summed squared residuals are shown for all simulated binding positions.

In Figure S9, summed squared residuals for selected binding positions (1, 7 and 12) are shown for all simulated PSFs. Minima are found at the assigned binding positions, and error bars correspond to the standard error of the mean. This demonstrates an unambiguous assignment to the respective simulated height range. However, the difference to neighboring positions can be quite small, which is most prominent directly at the tip (positions 1 - 4). This increases the probability for false height attribution in this range. We therefore estimate the accuracy of correct position attribution to be in the order of ± 10 nm. In the middle section of the nanocone, more pronounced minima are found, allowing for a more reliable classification, possibly down to single-digit nanometers.

References

1. M. Ringler et al., "Shaping Emission Spectra of Fluorescent Molecules with Single Plasmonic Nanoresonators," Phys. Rev. Lett. **100**(20), 203002 (2008) [doi:10.1103/PhysRevLett.100.203002].
